# Identification of Agents That Ameliorate Hyperphosphatemia-Suppressed Myogenin Expression Involved in the Nrf2/p62 Pathway in C2C12 Skeletal Muscle Cells

**DOI:** 10.3390/ijms232315324

**Published:** 2022-12-05

**Authors:** Shu-Man Hsieh Li, Shu-Ting Liu, Yung-Lung Chang, Gunng-Shinng Chen, Shih-Ming Huang

**Affiliations:** 1 Department of Biochemistry, National Defense Medical Center, Taipei City 114, Taiwan; 2 School of Dentistry, Department of Dentistry of Tri-Service General Hospital, National Defense Medical Center, Taipei City 114, Taiwan

**Keywords:** hyperphosphatemia, myogenin, nuclear factor erythroid 2-related factor 2, p62, L-ascorbic acid

## Abstract

Hyperphosphatemia can occur as a result of reduced phosphate (P_i_) excretion in cases of kidney dysfunction, which can induce muscle wasting and suppress myogenic differentiation. Higher P_i_ suppresses myogenic differentiation and promotes muscle atrophy through canonical (oxidative stress-mediated) and noncanonical (p62-mediated) activation of nuclear factor erythroid 2-related factor 2 (Nrf2) signaling. However, the crosstalk between myogenin and Nrf2/p62 and potential drug(s) for the regulation of myogenin expression needed to be addressed. In this study, we further identified that myogenin may negatively regulate Nrf2 and p62 protein levels in the mouse C2C12 muscle cell line. In the drug screening analysis, we identified N-acetylcysteine, metformin, phenformin, berberine, 4-chloro-3-ethylphenol, cilostazol, and cilomilast as ameliorating the induction of Nrf2 and p62 expression and reduction in myogenin expression that occur due to high P_i_. We further elucidated that doxorubicin and hydrogen peroxide reduced the amount of myogenin protein mediated through the Kelch-like ECH-associated protein 1/Nrf2 pathway, differently from the mechanism of high Pi. The dual functional roles of L-ascorbic acid (L-AA) were found to be dependent on the working concentration, where concentrations below 1 mM L-AA reversed the effect of high P_i_ on myogenin and those above 1 mM L-AA had a similar effect of high P_i_ on myogenin when used alone. L-AA exacerbated the effect of hydrogen peroxide on myogenin protein and had no further effect of doxorubicin on myogenin protein. In summary, our results further our understanding of the crosstalk between myogenin and Nrf2, with the identification and verification of several potential drugs that can be applied in rescuing the decline of myogenin due to high P_i_ in muscle cells.

## 1. Introduction

Serum phosphate (P_i_) levels are primarily regulated by the kidney, and hyperphosphatemia may occur because of reduced Pi excretion in kidney dysfunction [1,2]. Many studies have demonstrated that P_i_ is a pro-aging factor linked with muscle wasting [3,4,5]. Sarcopenia is a geriatric syndrome that involves an imbalance between anabolic and catabolic pathways for muscle mass [6,7]. Hyperphosphatemia could induce muscle wasting and suppress myogenic differentiation through oxidative stress-mediated activation of nuclear factor erythroid 2-related factor 2 (Nrf2) signaling [5]. However, the detailed molecular mechanisms underlying muscle atrophy induced by higher concentrations of P_i_ are not fully understood.

Excess reactive oxygen species (ROS) production could lead to muscle atrophy mediated through the inhibition of muscle protein synthesis and the promotion of protein breakdown [8]. Nrf2 is an important transcription factor that functions as a sensor of oxidative stress within eukaryotic cells [9,10]. Accumulation of ROS is linked to canonical activation of Nrf2 by preventing binding to its native repressor Kelch-like ECH-associated protein 1 (Keap1), which represses the transcriptional activity of Nrf2 through its sequestering, ubiquitination, and proteasomal degradation under basal conditions [10,11]. Administration of P_i_ entry blockade, cytosolic ROS scavenger, or Nrf2 inhibitors reverses the inhibitory effects of high P_i_ on myogenic differentiation. The transcriptional activity of Nrf2 on myogenin mRNA was verified to serve as a repressor. These data suggest that high P_i_ may be involved in both canonical and noncanonical activation of Nrf2 signaling pathways [5]. However, the functional interaction between Nrf2 and myogenin remains to be further addressed.

MyoD, MRF4, Myf5, and myogenin are members of the MyoD family of transcription factors [12,13]. Myogenin is a muscle-specific basic helix–loop–helix (bHLH) transcription factor involved in the coordination of skeletal muscle myogenesis and repair [14]. In muscle cells, the decline of endogenous myogenin proteins can lead to the reversal of muscle cell differentiation and the creation of mononucleated cells [15]. Myogenin cannot be functionally complemented by MyoD and Myf5 during differentiation [16,17]. Hence, it is important to understand the regulation of myogenin expression in the reversal of muscle cell differentiation resulting from exposure to exogenous stresses.

In our previous study, we demonstrated that high P_i_ suppressed myogenic differentiation in vitro and promoted muscle atrophy in vivo through canonical (ROS-mediated) and noncanonical (p62-mediated) activation of Nrf2 signaling [5]. We then demonstrated that Nrf2 repressed the expression of myogenin mRNA and induced the expression of p62 mRNA. Here, we further screened drugs that could disrupt Nrf2/p62 signaling involved in the expression of myogenin and compared different regulatory patterns of myogenin expression in a mouse C2C12 skeletal muscle cell model. In our work, we not only identified potential drugs to be applied in avoiding muscle dedifferentiation but also further clarified the regulatory mechanism of myogenin expression by P_i_, doxorubicin, hydrogen peroxide, and L-ascorbic acid (L-AA).

## 2. Results

### 2.1. Crosstalk of Myogenin with the Nrf2/p62 Complex and Other Proteins in High P_i_-Treated C2C12 Cells

**Figure 1:**
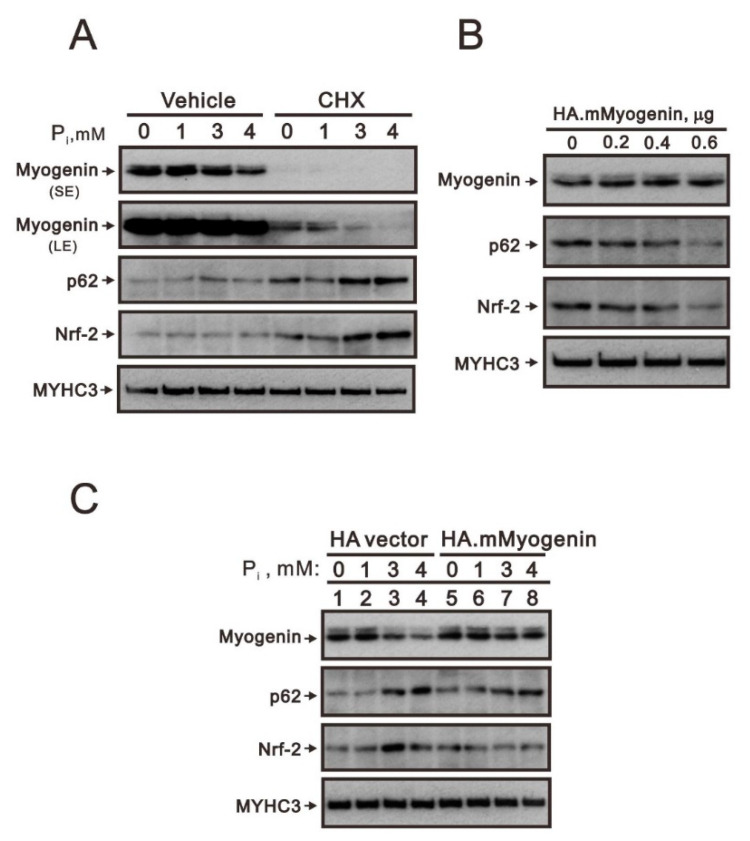
The effects of myogenin induced by high P_i_ on Nrf2 and p62 proteins in differentiated C2C12 cells. (**A**) After induction of differentiation by culturing C2C12 cells in differentiation medium for 3 days, differentiated C2C12 cells were pretreated for 2 h with CHX (5 μg/mL) and then were treated for 24 h with the indicated concentrations of P_i_. (**B**) Differentiated C2C12 cells were transiently transfected with indicated amounts of pSG5.HA. Myogenin for 4 h and further cultured for 20 h. (**C**) Differentiated C2C12 cells were transiently transfected with indicated amounts of pSG5.HA.Myogenin for 4 h and then treated with the indicated concentrations of P_i_ for 20 h. (**A**–**C**) Immunoblot analysis of myogenin, p62, Nrf2, and MYHC3 (myosin heavy chain isoform 3) levels. MYHC3 was a protein loading control. SE: shorter exposure; LE: longer exposure.

C2C12 cells, a subgroup of C2 myoblast, differentiate and fuse into a myotube when exposed to low-serum differentiation media [18]. These properties of C2C12 myotubes expressing contractile proteins and contracting spontaneously make C2C12 cells a tool for understanding the molecular mechanisms of muscle development. The limitation of C2C12 cells is the detachment of myotubes which subsequently leads to cell death. However, in our previous work in C2C12 cells, we demonstrated that high P_i_ could decrease myogenin gene and protein expression via the Nrf2/p62 pathway [5]. We confirmed that the induction of Nrf2 by high P_i_ was not primarily mediated through the Keap1/Nrf2 pathway as a response to ROS since proteasome inhibitor MG132 treatment was ineffective. It is a puzzle whether protein stability was involved in the regulation of myogenin. Hence, to clarify this possibility, we applied cycloheximide (CHX), an inhibitor of de novo protein synthesis (Figure 1A). The C2C12 cells treated with higher concentrations of P_i_ resulted in the suppression of myogenin protein stability. We observed the stability of myogenin was decreased with increasing concentrations of P_i_, whereas Nrf2 and p62 proteins were stabilized by higher concentrations of P_i_. Overexpression of myogenin proteins via a transient transfection strategy consistently suppressed the amounts of endogenous Nrf2 and p62 proteins in C2C12 cells, suggesting that myogenin might be involved in the negative effects on Nrf2 and p62 proteins (Figure 1B). In combination with high P_i_, transient overexpression of HA-myogenin in C2C12 cells reversed the effects of high P_i_ in the reduction of myogenin protein and the induction of Nrf2 and p62 proteins (Figure 1C).

**Figure 2:**
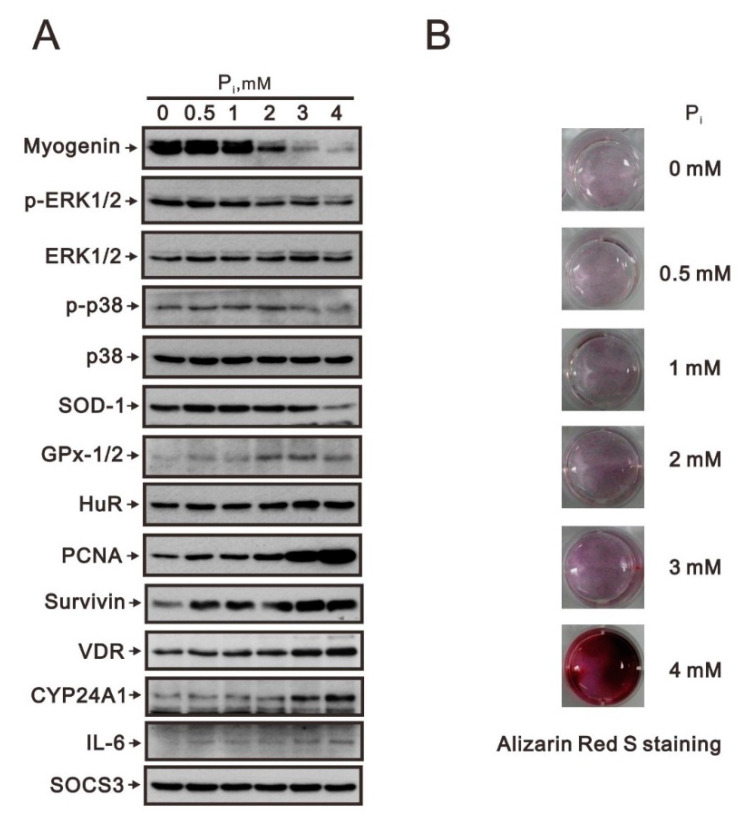
The effects of high P_i_ on myogenin and its associated proteins in differentiated C2C12 cells. After induction of differentiation by culturing C2C12 cells in differentiation medium for 3 days, differentiated C2C12 cells were treated with the indicated concentration of P_i_ for 24 h. (**A**) Cell lysates were subject to immunoblot analysis against indicated proteins. (**B**) Cells treated with P_i_ were stained with Alizarin Red S dye.

Myogenin is an important transcription factor for muscle terminal differentiation [12,15]. In addition to the suppression of myogenin protein expression by high P_i_ related to the Nrf2/p62 pathway, we further examined other proteins involved in the level of myogenin proteins. Here, we checked signaling pathways, such as ERK1/2 and p38, antioxidative enzymes (superoxide dismutase 1, SOD1, and glutathione peroxide-1/2, GPX-1/2), enzymes for calcitriol inactivation (cytochrome P450 24A1, cyp24A1), proteins involved in RNA binding (HuR), cellular proliferation (proliferating cell nuclear antigen, PCNA), survival (survivin), and inflammation (interleukin-6, IL-6). Our data showed that high P_i_ suppressed the ratio of p-ERK/ERK and p-p38/p38 and SOD1 in C2C12 cells (Figure 2A). We observed that high P_i_ increased GPX-4, HuR, PCNA, survivin, vitamin D receptor (VDR), cyp24A1, and IL-6 in C2C12 cells. No apparent effect on the suppressor of cytokine signaling 3 (SOCS3) protein was observed under the current conditions.

C2C12 is a pluripotent mesenchymal cell line for which osteoblast differentiation can be induced by bone morphogenetic protein-2 (BMP-2) [19,20]. Mature muscle can undergo a reversal of differentiation in the presence of high P_i_, which causes a decreased abundance of the muscle terminal differentiation transcription factor myogenin. Hence, we examined the differentiation status of C2C12 cells treated with different concentrations of P_i_ using the dye Alizarin Red S, which is a stain commonly used to identify calcium-containing osteocytes [21]. Our staining data showed that higher concentrations of P_i_ enhanced the Alizarin Red S staining in C2C12 cells (Figure 2B). Our data suggested that myogenin downregulation might have the ability to reverse the differentiation of horse serum-induced C2C12 muscle cell maturation into pluripotent mesenchymal cells, which then potentially trans-differentiate into osteocytes in response to higher concentrations of P_i_.

### 2.2. The Identification of Compounds Involved in the Regulation of Myogenin Expression via a Drug Screening Strategy with the Myogenin/Nrf2/p62 Platform

**Figure 3:**
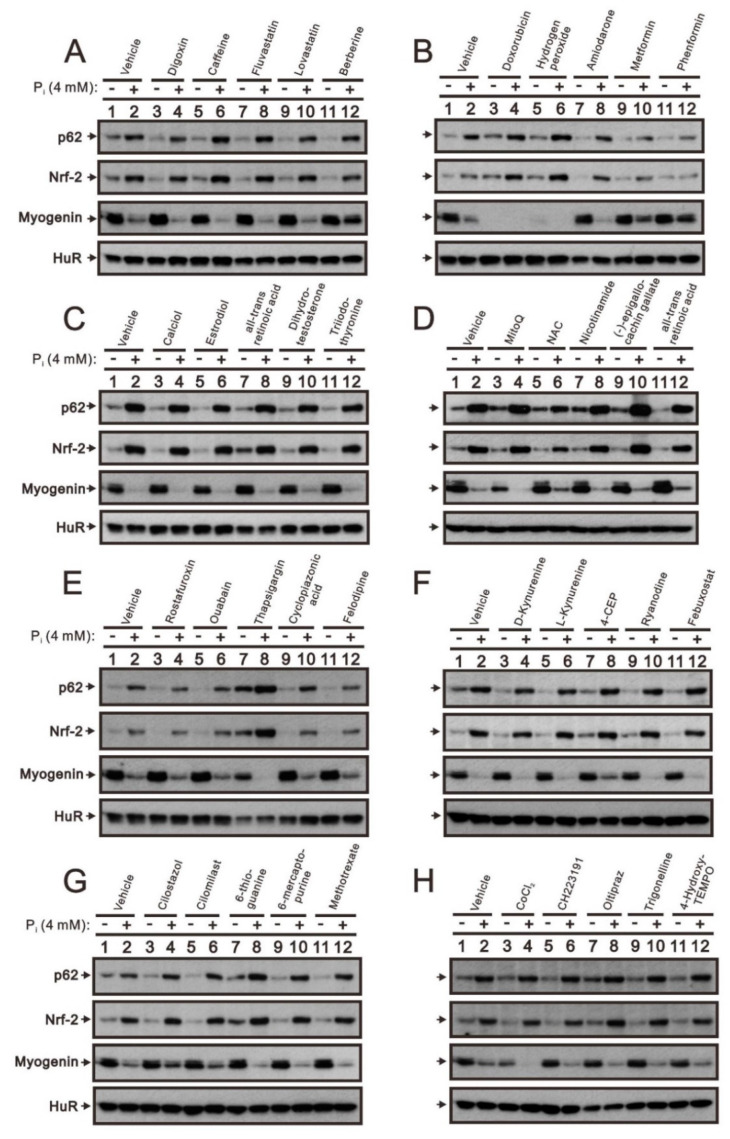
The drug screening platform for the rescue effect on the high P_i_ in differentiated C2C12 cells. After induction of differentiation by culturing C2C12 cells in differentiation medium for 3 days, differentiated C2C12 cells were treated with the indicated drug (**A**): vehicle, digoxin, caffeine, fluvastatin, lovastatin, and berberine; (**B**): vehicle, doxorubicin, hydrogen peroxide, amiodarone, metformin, and phenformin; (**C**): vehicle, calciol, estradiol, all-trans retinoic acid, dihydrotestosterone, and triiodothyronine; (**D**): vehicle, MitoQ, NAC, nicotinamide, (-)-epigallocachin gallate, and all-trans retinoic acid; (**E**): vehicle, rostafuroxin, ouabain, thapsigargin, cyclopiazonic acid, and felodipine; (**F**): vehicle, D-kynurenine, L-kynurenine, 4-CEP, ryanodine, and febuxostat; (**G**): vehicle, cilostazol, cilomilast, 6-thioguanine, 6-mercaptopurine, and methotrexate; (**H**): vehicle, CoCl_2_, CH223191, oltipraz, trigonelline, and 4-hydroxy-TEMPO) with 4 mM P_i_ for 24 h. Cell lysates were subject to immunoblot analysis against p62, Nrf2, and myogenin proteins. HuR was a protein loading control.

Our previous work provided evidence that phosphonoformic acid, N-acetylcysteine (NAC), metformin, and phenformin significantly diminish the stimulatory effect of high P_i_ on Nrf2 and p62 expression in addition to its inhibitory effect on myogenin expression [5]. We further examined the effects of 36 drugs on high P_i_-mediated Nrf2, p62, and myogenin expression (Figure 3). In addition to NAC, metformin, and phenformin, our screening data showed that berberine, 4-chloro-3-ethylphenol (4-CEP), cilostazol, and cilomilast ameliorate the induction of Nrf2 and p62 expression and reduction of myogenin expression caused by high P_i_. Thapsigargin and oltipraz reported effects similar to the above in the absence of high P_i_. Doxorubicin, hydrogen peroxide, MitoQ, and CoCl_2_ had direct negative effects on myogenin expression in C2C12 cells.

### 2.3. Comparison of the Effects of Doxorubicin, Hydrogen Peroxide, and L-Ascorbic Acid on the Regulation of Myogenin Expression in C2C12 Cells with High P_i_

**Figure 4:**
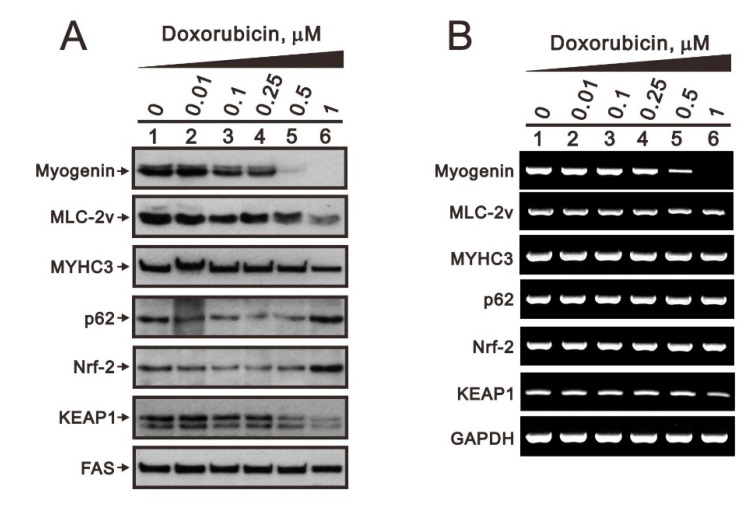
The effects of doxorubicin on the Keap1/Nrf2/p21/myogenin pathway in differentiated C2C12 cells. After induction of differentiation by culturing C2C12 cells in differentiation medium for 3 days, differentiated C2C12 cells were treated with the indicated concentration of doxorubicin for 20 h. (**A**) Cell lysates were subject to immunoblot analysis against myogenin, MLC-2v, MYHC3, p62, Nrf2, and Keap1 proteins. FAS was used as loading control. (**B**) Effects of doxorubicin on the mRNA expression of *myogenin*, *MLC-2v*, *MYHC3*, *p62*, *Nrf2*, and *Keap1* determined by RT-PCR. *GAPDH* was used as loading control.

We further examined the detailed mechanisms of doxorubicin and hydrogen peroxide (H_2_O_2_) regarding their effects on myogenin regulation related to the Nrf2/p62 pathway. We treated C2C12 cells with 0, 0.01, 0.1, 0.25, 0.5, and 1 µM doxorubicin for 20 h and then analyzed related proteins and mRNAs. Our Western blotting and RT-PCR analyses demonstrate that proteins and mRNA expression of myogenic factors, including myogenin, myosin light chain-2v (MLC-2v), and myosin heavy chain 3 (MYHC 3), were suppressed by doxorubicin in a dose-dependent manner (Figure 4A,B). However, p62 and Nrf2 proteins were initially suppressed and levels finally increased at the highest dosage, whereas their mRNA levels were unchanged. Examination of the well-known Keap1/Nrf2 pathway showed that Keap1 expression was suppressed by doxorubicin in a dose-dependent manner. Hence, we observed Keap1 downregulation at doxorubicin concentrations above 0.5 µM, whereby Nrf2 protein levels were accordingly increased.

**Figure 5:**
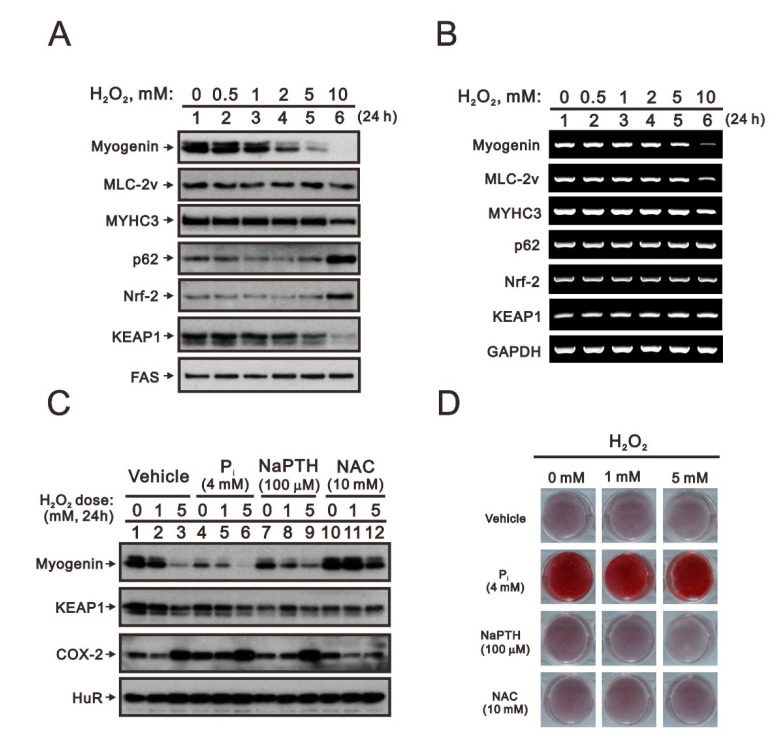
The effects of hydrogen peroxide on the Keap1/Nrf2/p62/myogenin pathway in differentiated C2C12 cells. (**A**,**B**) After induction of differentiation by culturing C2C12 cells in differentiation medium for 3 days, differentiated C2C12 cells were treated with the indicated concentration of hydrogen peroxide (H_2_O_2_) for 24 h. Cell lysates were subject to immunoblot analysis against indicated proteins. (**A**) Cell lysates were subject to immunoblot analysis against myogenin, MLC-2v, MYHC3, p62, Nrf2, and Keap1 proteins. FAS was used as loading control. (**B**) Effects of doxorubicin on the mRNA expression of *myogenin*, *MLC-2v*, *MYHC3*, *p62*, *Nrf2*, and *Keap1* determined by RT-PCR. *GAPDH* was used as loading control. (**C**,**D**) Differentiated C2C12 cells were treated with the indicated concentration of hydrogen peroxide with 4 mM P_i_, 100 μM NaPTH, or 10 mM NAC for 24 h. (**C**) Cell lysates were subject to immunoblot analysis against myogenin, Keap1, and Cox-2 proteins. HuR was a protein loading control. (**D**) Cells treated with indicated drugs were stained with Alizarin Red S dye.

C2C12 cells were treated with 0, 0.5, 1, 2, 5, and 10 mM H_2_O_2_ for 24 h, and the related proteins and mRNAs were then analyzed. Our Western blotting and RT-PCR analyses demonstrated that protein and mRNA expression of myogenic factors, including myogenin, MLC-2v, and MYHC3, were suppressed by H_2_O_2_ in a dose-dependent manner (Figure 5A,B). Similar to the doxorubicin response, p62 and Nrf2 proteins were initially suppressed and levels finally increased at the highest dosage, whereas mRNA levels were unchanged. Keap1 protein was suppressed by H_2_O_2_ in a dose-dependent manner, whereas *Keap1* mRNA was partially induced. We further combined H_2_O_2_ with 4 mM Pi, 100 µM sodium phosphonoformate tribasic hexahydrate (NaPTH), and 10 mM NAC to examine myogenin, Keap1, and Cox-2 levels using Western blotting analysis (Figure 5C). In C2C12 cells, H_2_O_2_ induced the expression of Cox-2 protein, a well-known H_2_O_2_ stress biomarker [22]. H_2_O_2_ suppressed the expression of Keap1 in a dose-dependent manner, suggesting that Nrf2 levels increase. Hence, the results in C2C12 cells confirmed that the suppression of myogenin expression was mediated through the Keap1/Nrf2 pathway. High P_i_ alone had positive effects on the expression of Cox-2, Keap1, and myogenin, and it potentiated the effect of H_2_O_2_ on the Keap1/Nrf2/myogenin pathway. A P_i_ transporter inhibitor, NaPTH, had negative effects on the expression of Keap1 and myogenin but not on the expression of Cox-2 protein. The application of a ROS scavenger, NAC, in isolation suppressed the expression of Keap1 but not of Cox-2 or myogenin. NAC was observed to totally suppress H_2_O_2_-induced Cox-2 expression and partially rescue H_2_O_2_-repressed myogenin expression in C2C12 cells. Except for in the presence of 4 mM P_i_, no changes in Alizarin Red S staining were observed regardless of whether cells were in H_2_O_2_-repressed myogenin expression conditions or otherwise (Figure 5D).

**Figure 6:**
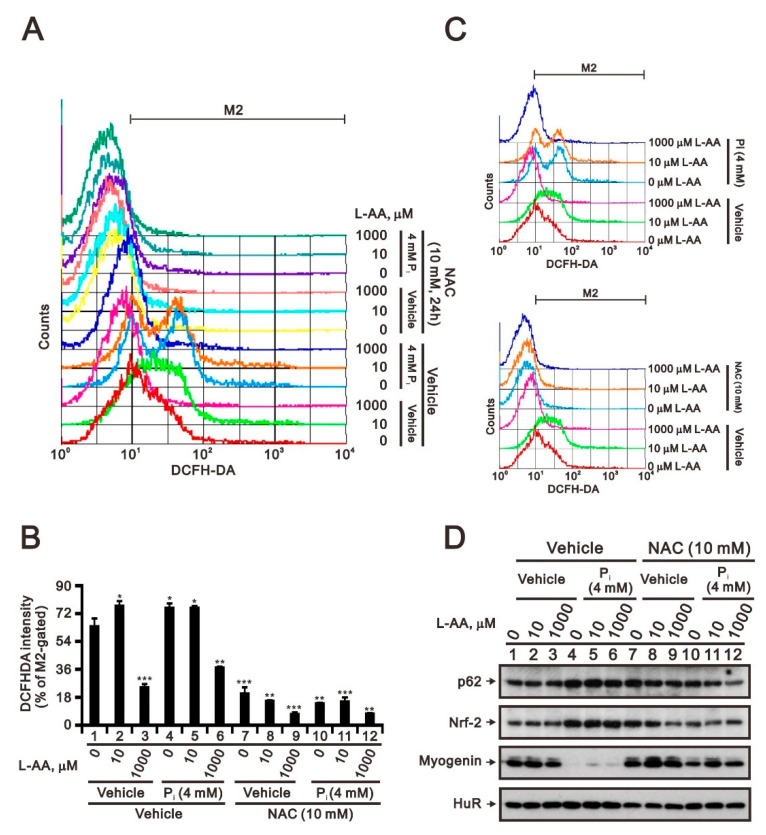
The effects of L-ascorbic acid on high P_i_-induced ROS generation in differentiated C2C12 cells. (**A**) Differentiated C2C12 cells were treated with the indicated concentrations of L-ascorbic acid (L-AA) combined with 5 mM NAC or 4 mM P_i_ for 24 h. (**A**,**B**) Cytosolic ROS levels were determined based on H2DCFDA fluorescence using the FL1-H channel: the x-axis represents H2DCFDA intensity; the y-axis indicates the cell counts at the corresponding fluorescence intensity. Bars depict the mean ± SD. * *p* < 0.05; ** *p* < 0.01; *** *p* < 0.001. (**C**) Bar graph summarizing the data from panel A as percentages of cells with increased ROS production (% M2 gated). (**D**) Cell lysates were subject to immunoblot analysis against p62, Nrf2, and myogenin proteins. HuR was used as loading control.

L-ascorbic acid (L-AA) has been reported as either an antioxidant or prooxidant depending on the applied cellular concentration [23,24]. Our data showed that antioxidant NAC has the ability to rescue the repression of myogenin expression by high P_i_ in C2C12 cells [5]. Hence, we applied various concentrations of L-AA to examine whether it had the ability to rescue the repression of myogenin expression by high P_i_ in C2C12 cells. We first measured the status of cytosolic ROS in the presence of high P_i_, NAC, L-AA, and NAC combined with L-AA using the dye DCFH-DA (Figure 6A–C). Our data showed that 1000 μM L-AA reduced cytosolic ROS, suppressed high P_i_-induced ROS, and synergistically worked with NAC to reduce cytosolic ROS and high P_i_-induced ROS in C2C12 cells. According to Western blotting analysis, L-AA had no apparent effect on the expression of p62, Nrf2, and myogenin at the tested concentrations (Figure 6D, compared with lanes 1–3). Moreover, L-AA did not appear to suppress the effects of high P_i_ regarding the expression of p62, Nrf2, and myogenin (Figure 6D, compared with lanes 4–6). Thus, L-AA appeared to synergistically function with NAC to suppress the effects of high P_i_ on the expression of p62, Nrf2, and myogenin proteins (Figure 6D, compared with lanes 7–12).

**Figure 7:**
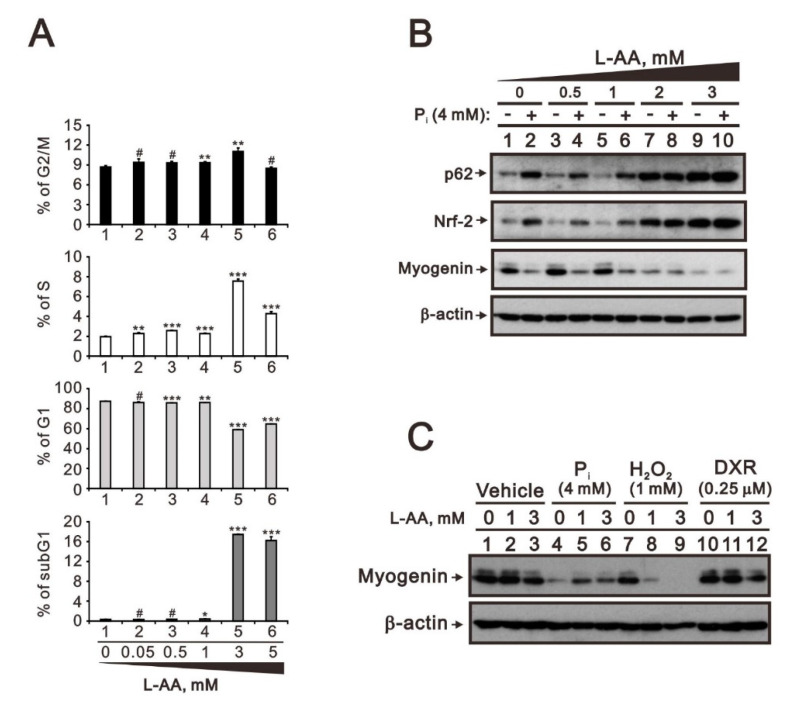
The effects of L-AA on the cell cycle profile and the crosstalk with P_i_, doxorubicin, and hydrogen peroxide in differentiated C2C12 cells. (**A**) Differentiated C2C12 cells were treated with the indicated concentrations of L-AA for 24 h. The cell cycle profile was analyzed with PI dye. Data are presented as a percentage of the control. Bars depict the mean ± SD. # *p* > 0.05; * *p* < 0.05; ** *p* < 0.01; *** *p* < 0.001. (**B**) Differentiated C2C12 cells were treated with the indicated concentrations of L-AA combined with 4 mM P_i_ for 24 h. Cell lysates were subject to immunoblot analysis against p62, Nrf2, and myogenin proteins. β-actin was used as loading control. (**C**) Differentiated C2C12 cells were treated with the indicated concentrations of L-AA combined with 4 mM P_i_, 1mM hydrogen peroxide (H_2_O_2_), or 0.25 µM doxorubicin (DXR) for 24 h. Cell lysates were subject to immunoblot analysis against myogenin protein. β-actin was used as loading control.

We further elevated the concentration of L-AA up to 5 mM to examine its effect on C2C12 cells using cell cycle profile analysis (Figure 7A). We observed a higher population of subG1 phase cells at concentrations higher than 3 mM, accompanied by the downregulation of G1 populations and the upregulation of S and G2/M populations. The cell cycle profiling results suggested that 5 mM L-AA is harmful to C2C12 cells and affects their survival. Hence, we selected 3 mM L-AA as the highest concentration for Western blotting analysis. Over 2 mM L-AA alone increased the expression of p62 and Nrf2 proteins and decreased the expression of myogenin proteins to the same degree as high P_i_ (Figure 7B). It was difficult to examine the functional interaction between high P_i_ and L-AA on p62, Nrf2, and myogenin proteins under this condition. The comparison between L-AA and high P_i_ treatments suggested that higher L-AA concentrations as well as high P_i_ increased the expression of Nrf2 and p62 via ROS induction. Higher concentrations of L-AA partially rescued high P_i_-repressed myogenin proteins (Figure 7C, compared with lanes 4–6) and further enhanced the suppressive effects of H_2_O_2_ in repressing myogenin proteins in C2C12 cells (Figure 7C, compared with lanes 7–9).

## 3. Discussion

Hyperphosphatemia can induce muscle wasting and suppress myogenic differentiation [1,2,4]. Our recent study demonstrated that the treatment of differentiating C2C12 cells with P_i_ induced protein degradation and reduced protein synthesis in a dose-dependent manner [5]. Treatment with either P_i_ influx, cytosolic ROS, or Nrf2 inhibitors abrogated the inhibitory and stimulatory effect of high P_i_ on myogenin and Nrf2 and p62 expression, respectively. Nrf2 could serve as a repressor of myogenin and activator of p62 transcription. High P_i_ concentrations disrupt the mitochondrial membrane potential and oxidative phosphorylation in differentiating C2C12 cells. These data demonstrate that hyperphosphatemia suppresses skeletal muscle cell differentiation through induction of oxidative stress, activation of the Nrf2/p62 signaling pathway, and disruption of mitochondrial function. In this study, we further identified that myogenin might negatively regulate Nrf2 and p62 at the protein level. In the drug screening analysis, we identified NAC, metformin, phenformin, berberine, 4-CEP, cilostazol, and cilomilast as significantly diminishing the stimulatory effect of high P_i_ on Nrf2 and p62 expression as well as its inhibitory effect on myogenin expression. In our work, we further elucidated that doxorubicin and hydrogen peroxide reduce the amount of myogenin protein, which is mediated through the Keap1/Nrf2 pathway and different from the mechanism of high P_i_. The dual functional roles of L-AA were dependent on the working concentration, where concentrations below 1 mM L-AA reversed the effect of high P_i_ on myogenin and those above 1 mM L-AA, applied in isolation, had an effect similar to that of high P_i_ on myogenin. L-AA exacerbated the effects of hydrogen peroxide on myogenin protein and did not influence the effect of doxorubicin on myogenin protein.

There is growing evidence for the association of drugs with muscle, where they may act as a trigger in the development of sarcopenia and frailty. Sarcopenia is a geriatric syndrome involving an imbalance between the anabolic and catabolic pathways that regulate muscle mass [4,6,7]. Innovative anticancer strategies have contributed to improving the survival of cancer patients, transforming cancer into a chronic rather than terminal disease, and include the use of doxorubicin, with its well-known cardiotoxicity, which results in myocardial fibrosis and necrosis via ROS production [25,26]. Myopathy refers to diseases that affect skeletal muscles. Myopathies are usually degenerative, but they are sometimes caused by drug side effects, electrolyte imbalance, or chronic immune system disorders [27,28]. For chronic kidney disease patients, hyperphosphatemia is one of the side effects of long-term hemodialysis [1,2]. Our current study focuses on high P_i_ and highlights the protein–protein interaction between myogenin and Nrf2 via the modulation of ROS, which can serve as potential therapeutic targets for developing drugs reducing the risks associated with hemodialysis [5].

These myopathies are often related to a drug’s ability to alter the balance of metabolism and protein, induce necrosis, or impair autophagy [28]. In our drug-screening results, we identified many potential drugs, including metformin and phenformin. The effects of metformin on muscle are still uncertain and, therefore, a matter of debate [29,30], because metformin use does not alter mTOR expression, thus appearing to negate this theory [31]. The use of metformin in preventing the development of sarcopenia in older people with prediabetes is supported by the results of a clinical trial [32]. Vitamin D is related to muscle strength and frailty, which can be either dependent or independent of binding to its receptor, VDR [33,34,35]. Vitamin D supplementation is demonstrated to have beneficial effects in increasing muscle strength and performance [35]. Here, our screening data failed to support the role of vitamin D in rescuing the regulation of myogenin via the Nrf2/p62 pathway. However, the induction of VDR expression by high P_i_ provides further issue in addressing the functional role of vitamin D/VDR and muscle differentiation. The nuclear localization of VDR as a transcription factor is more important than the degree of expression. Hence, we will address the transcriptional function of vitamin D/VDR in the presence of high P_i_ in the future.

In addition to myogenin, retinoblastoma protein was identified as prompting the reversal of differentiation in terminally differentiated muscle cells [36]. The Alizarin Red S dye is a commonly used stain to identify calcium-containing osteocytes [21]. P_i_ acts as an intracellular signaling molecule to regulate the expression of numerous osteogenic genes, including osteopontin and BMP-2 [37]. C2C12 is a pluripotent mesenchymal cell line for which osteoblast differentiation can be induced by BMP-2 [19,20]. In this study, we applied this dye to determine whether the decline of myogenin could reverse the differentiation status of C2C12 cells. In such a scenario, terminally differentiated C2C12 cells might be dedifferentiated into immature muscle cells or pluripotent mesenchymal progenitor, which might then differentiate into osteocytes in response to high P_i_. Our current data imply two possibilities, one in which high P_i_ can cause differentiation of myogenin-deficient C2C12 cells into osteocytes, and the other where high P_i_ causes misleading responses of Alizarin Red S. Without the solid evidence of osteocytic protein expression, we could not rule out the crosstalk between P_i_ and calcium in the presence of a higher concentration of exogenous P_i_. We failed to observe a positive response of Alizarin Red S staining similar to the effect of hydrogen peroxide on myogenin expression. One study demonstrated that activation of ERK and p38 are both essential components in BMP-2 signaling that lead to the induction of the osteoblast phenotype in C2C12 cells [38]. The activation of the ERK signaling pathway by P_i_ is biphasic, whereby the two phases are separated by a time interval of several hours. The second phase of ERK activation is required for gene regulation in cases where the first alone is insufficient. In our current data, high P_i_ decreased the activation of the ERK signaling pathway in C2C12 cells, suggesting that exogenous high P_i_ disfavors the osteoblastic commitment process in our study condition.

L-AA induces hydrogen peroxide formation at pharmacologic concentrations (>100 μM) achieved by intravenous administration [24,39,40,41], in contrast to physiological concentrations that neutralize the damage of ROS. In our study, physiologic concentration L-AA failed to serve as a ROS scavenger as NAC for the relief of P_i_ effects on C2C12 cells, whereas pharmacological concentration L-AA exacerbated the effects of P_i_ in C2C12 cells. Our Figure 5C data showed that NAC had the ability to rescue the effect of hydrogen peroxide on myogenin, but pharmacological concentration L-AA exacerbated this effect (Figure 7C). In normal cells, hydrogen peroxide can initiate Fenton’s reactions and cause oxidative damage to cellular macromolecules [42,43,44]. The recycling of endogenous ascorbate enables continuous production of hydrogen peroxide which supports pharmacological concentration L-AA exacerbated the effects of P_i_ on C2C12 cells in this study. Many studies demonstrated that the steady-state levels of ROS stress in cancer cells are higher than in normal cells and L-AA kills some tumor cells via hydrogen peroxide-related mechanisms but not normal cells [45,46]. In addition to the involvement of redox homeostasis, L-AA can also serve as a cofactor for the Fe^2+^-2-oxoglutarate-dependent dioxygenase family for the stability of hypoxia-inducible factor 1 alpha (HIF-1α) and ten-eleven translocation 2 (Tet2) DNA hydroxylases, which catalyze the demethylation of modified DNA [40,41,47,48,49]. Hence, the applications of L-AA as a repurposing drug for the modulation of the redox homeostasis involved in ROS and hydrogen peroxide productions and the enzymatic activity of dioxygenase involved in HIF-1α and Tet2 protein stabilities remain highlight potential

## 4. Materials and Methods

### 4.1. C2C12 Myotube Culture and P_i_ Treatment

Mouse C2C12 myoblasts seeded into 6-well plates at a density of 2 × 10^5^ cells/well were first cultured for 3 days in DMEM/HG (Dulbecco’s Modified Eagle’s Medium/high glucose (4500 mg/L)) supplemented with 10% fetal bovine serum (FBS) (Thermo Fisher Scientific, Waltham, MA, USA) at 37 °C under a 5% CO_2_. They were then cultured for an additional 4 days in differentiation medium consisting of DMEM/HG supplemented with 2% adult horse serum (HS) (Thermo Fisher Scientific). Sodium phosphate buffer (1 M Na_2_HPO_4_/NaH_2_PO_4_ (pH 7.4)) was then added to achieve the final Pi concentrations, and the C2C12 myotubes were incubated for 24 h.

### 4.2. Immunoblotting

C2C12 cells were lysed in RIPA lysis buffer (50 mM HEPES (pH 7.6), 150 mM sodium chloride, 0.5% Triton X-100, 10% glycerol, and 0.1% dodecyl sulfate) for 10 min at 4 °C. The resultant protein extracts (30 μg) were separated by 12% SDS-PAGE at 80 V and electro-transferred onto PVDF membrane (Immobilon-P; Millipore, Bedford, MA, USA) using a semi-dry Trans-Blot Turbo System (Bio-Rad, Hercules, CA, USA) at 25 V, 1.0 A for 30 min. To check transfer efficiency, Ponceau S staining was performed on PVDF membranes. The membrane was blocked with 5% nonfat milk in PBS with 0.01% Tween-20 (TBS-T) for 1 h and then incubated with diluted primary antibodies (Table 1) at 4 °C overnight with shaking. The membrane was washed in TBS-T for 15 min and repeated three times and incubated with HRP-conjugated secondary antibodies at room temperature for 1 h. The membrane was then washed in TBS-T for 15 min and repeated three times. The immunoreactive proteins were detected using ECL^TM^ Western Blotting Detection Reagent and Amersham Hyperfilm^TM^ ECL (GE Healthcare, Waukesha, WI, USA).

### 4.3. Alizarin Red S Staining

Mouse C2C12 myoblast cells seeded into 12-well culture plates at a density of 1 × 10^5^ cells/well were first cultured for 3 days in DMEM/HG supplemented with 10% FBS at 37 °C under 5% CO_2_ and then cultured for an additional 4 days in differentiation medium consisting of DMEM/HG supplemented with 2% HS. The spindle-shaped C2C12 myoblast cells then differentiated and fused, gradually forming multinucleate myotubes. Sodium phosphate buffer (1M Na_2_HPO_4_/NaH_2_PO_4_, pH 7.4) was then added to the indicated Pi concentrations of 0, 0.5, 1, 2, 3, or 4 mM or treated with H_2_O_2_ dose of 1 and 2 mM combined with P_i_ (4 mM), NaPTH (100 μM), or NAC (10 mM), and C2C12 myotubes were incubated for 24 h. After treatment, the medium was carefully aspirated, and the cells were washed with Dulbecco’s PBS w/o Ca^2+^/Mg^2+^ twice. After carefully aspirating the PBS, the cells were fixed by incubation in 10% formalin for at least 30 min. After fixation, the formalin was aspirated, and the cells were washed with distilled water twice. The distilled water was then carefully aspirated, and the cells were stained with Alizarin Red S staining solution (Alizarin, 94%, A14404, Alfa Aesar (Ward Hill, MA, USA); the Alizarin Red S staining solution was prepared by dissolving 2 g powder in 100 mL distilled water, mixed, and pH adjusted to 4.1~4.3 with HCl or NH_4_OH) with incubation at room temperature in the dark for 45 min. After staining, the cells were washed with distilled water four times and PBS added to analyze the cells (calcium deposits are specifically stained bright orange-red by Alizarin Red S).

### 4.4. Flow Cytometric Analyses of Cell Cycle Profile and ROS Generation

The cell cycle profile was determined by measuring the DNA content of cells using fluorescence-activated cell sorting (FACS). Differentiated C2C12 cells were fixed in 70% ice-cold ethanol and kept at 20 °C overnight. Before analysis, the harvested C2C12 cells were washed twice with ice-cold PBS and stained with propidium iodide (PI) (5 μg/mL PI in PBS, 0.5% Triton X-100, and 0.5 μg/mL RNase A) for 30 min at room temperature in the dark. All the samples were analyzed using a FACSCalibur flow cytometer (BD Biosciences, San Jose, CA, USA). The data were analyzed using the Cell Quest Pro software (BD Biosciences).

Cytosolic ROS levels were measured using the fluorescent indicator H2DCFDA (2′,7′-dichlorodihydrofluorescein diacetate). Myotubes treated for 24 h with the indicated concentrations of P_i_ were stained with 10 μM H2DCFDA at 37 °C. After incubation for 30 min, ROS levels were assessed using a FACSCalibur flow cytometer (BD Biosciences). The excitation and emission wavelengths were 488 and 525 nm, respectively.

### 4.5. Reverse Transcription-Polymerase Chain Reaction (RT-PCR)

Total RNAs were isolated from differentiated C2C12 cells using TRIzol reagent (Invitrogen, Waltham, MA, USA). Reverse transcription for first-strand cDNA synthesis was carried out using MMLV reverse transcriptase (Epicentre Biotechnologies, Madison, WI, USA) with 1 μg of total RNA at 37 °C for 60 min. Specific primers (Table 2) were designed and evaluated by the program “Pick Primers” on the National Center for Biotechnology Information website (https://www.ncbi.nlm.nih.gov/tools/primer-blast/) (accessed on 1 December 2021). Primers, dNTP, and Taq DNA polymerase were added for subsequent PCR reactions, which were processed using a Veriti™ Simpli Thermal Cycler (Applied Biosystems, Foster City, CA, USA).

### 4.6. Statistical Analyses

The values are expressed as the mean ± SD of at least three independent experiments. All the comparisons between groups were conducted using unpaired two-tailed *t*-tests. The statistical significance was set at *p <* 0.05.

## 5. Conclusions

Our data demonstrate that myogenin might negatively regulate Nrf2 and p62 at the protein level, and Nrf2 is a repressor of myogenin expression. In addition to NAC, metformin, and phenformin, berberine, 4-CEP, cilostazol, and cilomilast were identified to diminish the induction of Nrf2 and p62 expression and the reduction of myogenin expression by high P_i_ in the drug screening analysis. Doxorubicin and hydrogen peroxide reduced the amount of myogenin protein mediated through the Keap1/Nrf2 pathway, different from the mechanism of high P_i_. The dual functional roles of L-ascorbic acid were dependent on the working concentration, where concentrations below 1 mM L-AA reversed the effects of high P_i_ on myogenin and those above 1 mM L-AA, applied in isolation, had effects similar to those of high P_i_ on myogenin. L-AA exacerbated the effect of hydrogen peroxide on myogenin protein and did no influence the effect of doxorubicin on myogenin protein.

## Figures and Tables

**Table 1:** Antibodies used for this study.

Santa Cruz Biotechnoglog (Santa Cruz, CA, USA)		
MYHC3	sc-376157	1:2000
SOD-1	sc-101523	1:1000
GPx-1/2	sc-133160	1:1000
beta-actin	sc-47778	1:10,000
HuR	sc-5261	1:2000
Nrf-2	sc-365949	1:1000
p62	sc-28359	1:1000
PCNA	sc-25280	1:2000
VDR	sc-13133	1:1000
CYP24A1	sc-365700	1:1000
SOCS3	sc-51699	1:1000
FAS	sc-55580	1:1000
COX-2	sc-1745	1:1000
**Abcam (Trumpington, C** **ambridg, UK)**		
Myogenin	ab124800	1:2000
Survivin	ab76424	1:1000
**Cell signaling Biotechnology (** **T** **opsfield, MA, USA)**		
p-ERK1/2	#4370	1:1000
ERK1/2	#4695	1:1000
p-p38	#9212	1:1000
p38	#9211	1:1000
IL-6	#12153	1:1000
MLC2v	#8505	1:1000
**Proteintech Group (Rosemont, IL, USA)**		
Keap1	60027-I-Ig	1:1000

**Table 2:** PCR primers used for this study.

Gene Name	Sequence (5′→3′)
*Myogenin*	Forward: 5′-CTACCTTCCTGTCCACCTTC-3′
	Reverse: 5′-CTCCAGTGCATTGCCCCACT-3′
*MYHC3*	Forward: 5′-GCCTCATCCACACCAAGAAGA-3′
	Reverse: 5′-TCCACCAGATCCTGCAATCTC-3′
*MLC-2v*	Forward: 5′-CTCCAACGTGTTCTCCATG-3′
	Reverse: 5′-AGTCCTTCTCTTCTCCGTGGG-3′
*p62*	Forward: 5′-GTGATGAGGAGCTGACAATGG-3′
	Reverse: 5′-TGGAGCAGAAGCTGACTCAG-3′
*Nrf-2*	Forward: 5′-GAGAATTCCTCCCAATTCAGC-3′
	Reverse: 5′-ACCATGAAGGAAATGTGGACC-3′
*KEAP1*	Forward: 5′-CACACTAGAGGATCACACCAAG-3′
	Reverse: 5′-GCTGGTGCAGTTCAGTGCAG-3′
*GAPDH*	Forward: 5′-CTTCATTGACCTCAACTAC-3′
	Reverse: 5′-GCCATCCACAGTCTTCTG-3′

## Data Availability

Not applicable.
